# Supplementary material for: Copy number evolution in simple and complex tandem repeats across the C57BL/6 and C57BL/10 inbred mouse lines
Source: G3 (Bethesda). 2021 May 27;11(8):jkab184. doi: 10.1093/g3journal/jkab184 (PMC8496272; doi:10.1093/g3journal/jkab184)
Supplement: jkab184_Supplementary_Data [file jkab184_supplementary_data.zip › Supplemental/Supplementary_Figures.pdf]

Figure S1

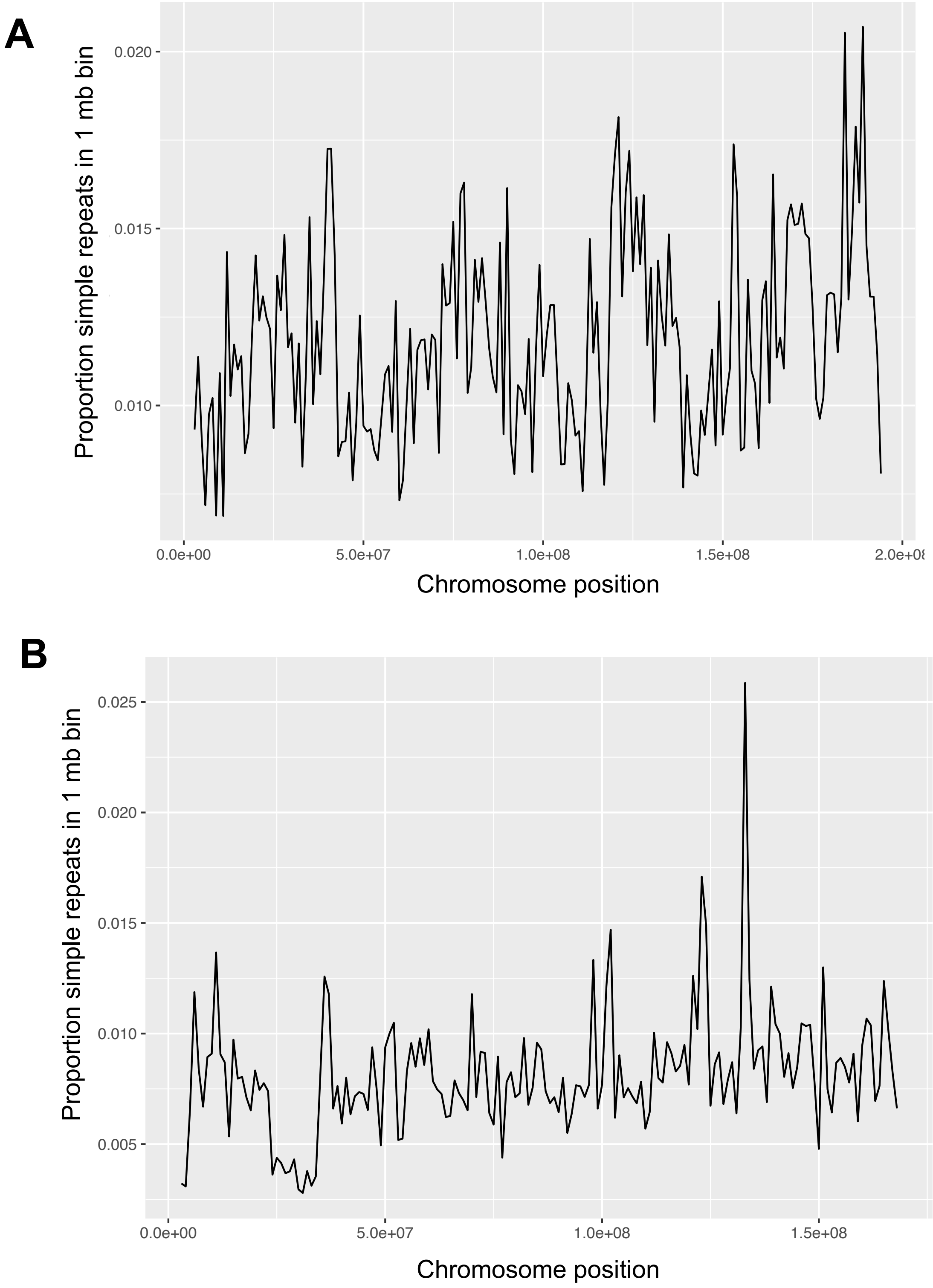

Figure S1. Density of simple repeats on mouse 39 assembly Chr1 (A) and ChrX (B). We used phobos to find simple repeats of 1-20 bp unit lengths in the assembly. We then filtered repeat arrays to only contain the 434 kmers we found to have at least 10 copies in one line with k-Seek. We only considered arrays that were at least 100 bp long. We then calculated the proportion of each 1 Mb bin across the chromosome that is made up of simple repeats.

Figure S2

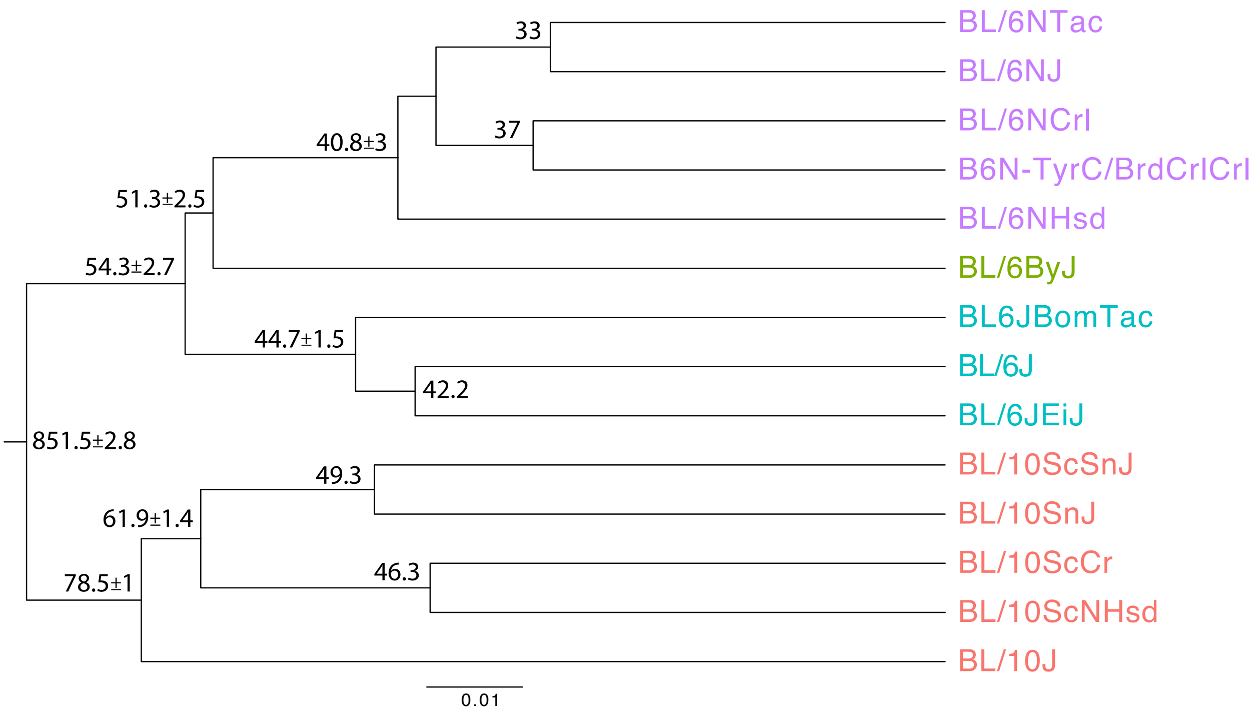

Figure S2. Phylogenetic reconstruction from SNP data. Variants were called with GATK and the R package SNPRelate was used to calculate a distance matrix based on identity-by-state, followed by hierarchical clustering and producing a tree. The SNP data was able to distinguish the B6 and B10 clades, but not all relationships within them. Branch length estimates in generations are written at each node. We used the tree produced here to estimate the branch length between B6 and B10 as ~850 generations.

Figure S3

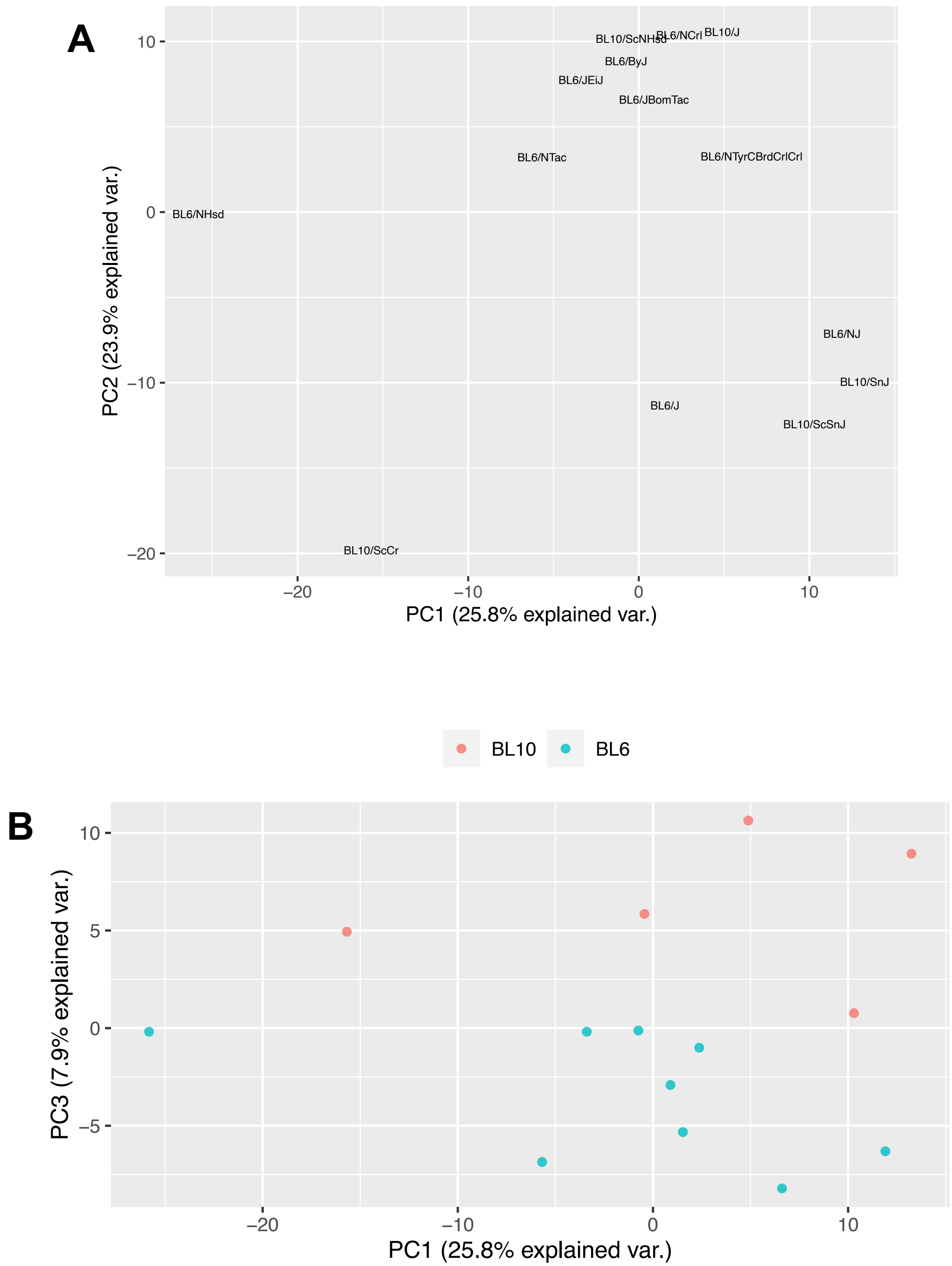

Figure S3. Principal components analysis (PCA) of kmer abundances. A) PC1 and PC2 do not show separation between the two major clades BL6 and BL10. B) PC1 and PC3; there is some grouping of BL6 and BL10 clades on PC3.

Figure S4

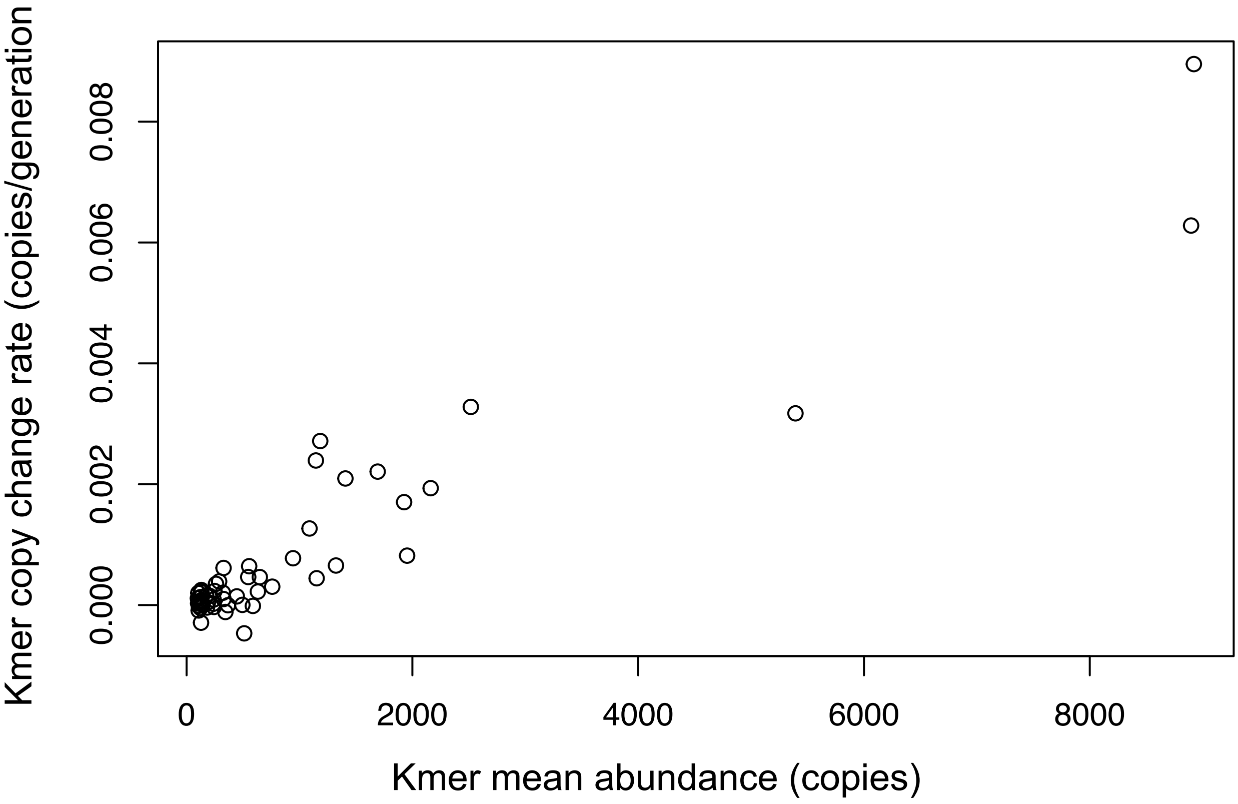

Figure S4. The copy number change rate is positively correlated with the kmer's mean abundance. The 6 most abundance kmers were removed from the plot to reduce skewing.
